# Longitudinal investigation of a single variant SARS-CoV-2-outbreak in the immunologically naïve population of Ulvik, Norway

**DOI:** 10.1186/s12879-024-09856-2

**Published:** 2024-10-15

**Authors:** Nicolay Mortensen, Knut-Arne Wensaas, Unni Solem, Audun Sivertsen, Harleen M. S. Grewal, Guri Rortveit, Elling Ulvestad, Sverre Litleskare

**Affiliations:** 1 Department of Clinical Science, Faculty of Medicine, University of Bergen, Bergen, Norway; 2 Department of Microbiology, Haukeland University Hospital, Bergen, Norway; 3 Norwegian Advisory Unit for Antibiotic Use in Hospitals, Bergen, Norway; 4 Research Unit for General Practice, NORCE Norwegian Research Centre, Bergen, Norway; 5 Ulvik municipality, Ulvik, Norway; 6 Department of Clinical Science, Bergen Integrated Diagnostic Stewardship Cluster, Faculty of Medicine, University of Bergen, Bergen, Norway; 7 Department of Global Public Health and Primary Care, University of Bergen, Bergen, Norway; 8 Norwegian Institute of Public Health, Oslo, Norway

**Keywords:** COVID-19, SARS-CoV-2, Complete outbreak, Long-term symptoms, Fatigue, Insomnia

## Abstract

**Purpose:**

To perform an extensive investigation of the clinical features and long-term complications among the *n* = 134 adults and children with nucleic acid amplification test (NAAT) verified SARS-CoV-2-infection in the immunologically naïve population of Ulvik, Norway, during the single variant B.1.1.7 outbreak in late January through February 2021.

**Methods:**

Every infected person regardless of whether symptoms of COVID-19 were present was invited to answer a web-based questionnaire at two- and seven months after testing positive. The period from initial infection to the first questionnaire was assessed retrospectively, and the time points at two- and seven months were assessed prospectively.

**Results:**

A total of 87 of 134 (65%) NAAT-positive persons answered the first questionnaire, of which 35/87 (40%) were children, and 74 of 87 (85%) answered the second questionnaire. Children experienced symptoms less often than adults during the acute phase of infection (51% (18/35) versus 81% (42/52) (*p* = .004)). At two-months follow-up 88% (53/60) of participants with symptoms during the acute phase, including all children, reported no longer having symptoms. Among those with persisting symptoms at seven months, fatigue (18/25) and insomnia (16/24) were common.

**Conclusion:**

In an immunologically naïve population infected with the SARS-CoV-2 B.1.1.7 variant, the clinical features of acute phase symptoms were similar to previous studies. Children underwent asymptomatic infection more often than adults, and adults more often experienced persisting symptoms. Insomnia and fatigue were common complaints among those with persisting symptoms seven months after infection.

**Supplementary Information:**

The online version contains supplementary material available at 10.1186/s12879-024-09856-2.

## Background

The first cases of Coronavirus Disease-2019 (COVID-19) were identified in December 2019 in Wuhan, China. COVID-19 is caused by the severe acute respiratory syndrome coronavirus 2 (SARS-CoV-2) which proved to be highly contagious and rapidly emerged as a major health crisis all over the world. On March 11th, 2020, the World Health Organization (WHO) declared COVID-19 a pandemic [1].

The presenting symptoms commonly include cough, dyspnea, fever, and fatigue. Severe illness is more likely in elderly patients and in individuals with prior illness [2]. A proportion of the infected patients experience complications that last beyond the typical convalescence period. Some of these are diagnosed with post-acute sequelae of COVID-19, also referred to as the long COVID syndrome or post COVID, of which the most common symptoms are fatigue, shortness of breath, sleep disturbances, loss of smell and taste, and cognitive dysfunctions. The reported prevalence of post-acute sequelae varies between studies, ranging from 10 to 70% of infected individuals [3, 4]. This wide range could reflect heterogeneity in the methods for symptom assessment, population characteristics (age, comorbidity, vaccine status, etc.), and in the strains of viruses in circulation at the given time [5].

From January through February 2021, there was an outbreak of the SARS-CoV-2 Variant of Concern B.1.1.7 in the small municipality of Ulvik, Norway. This occurred before the general population had access to vaccine. Prior to the outbreak there had been only four cases of COVID-19 in this community. Thus, the population could be considered immunologically naïve. In accordance with national guidelines an extensive regimen with expanded social distancing practices, case identification, testing and contact-tracing regimen was established. 13% (134/1061) of the total population was infected, with 30% being quarantined [6].

The aim of the current study is to characterize the infected population in Ulvik during the outbreak in detail and assess their symptom burden and long-term complications.

## Materials and methods

### Study setting and design

Ulvik is a rural municipality in the innermost part of the Hardanger fjord in Western Norway. It had 1061 inhabitants at the time of the outbreak [7], a single nursing home, one kindergarten, and a primary and secondary school.

The outbreak commenced on January 29th 2021 when the first case was detected. The sampling frequency of nasopharyngeal and throat swabs combined with contact-tracing was ramped up, and testing was freely available to all inhabitants regardless of symptoms. All symptomatic individuals and those who had been in close contact with SARS-CoV-2 positive persons were specifically encouraged to be tested. A total of 809 tests were performed on 554 individuals between January 29th and the outbreak’s culmination on February 22nd. Samples were analyzed by nucleic acid amplification testing (NAAT) at Haukeland University Hospital. The B.1.1.7 variant was detected in all positive samples. Virus evolution through the transmission network as well as household secondary attack rates have been explored in detail in a previous study [8].

This was an observational study where all 134 persons with a positive NAAT were invited to answer a web-based questionnaire two months after testing positive, which was the time that it took to obtain ethical approval and develop the research protocol. Those who responded after two months were invited to the seven-months follow-up.

### Definition of study time periods and corresponding data sources

The data from the two questionnaires were conceptually divided into three time periods: The acute phase of infection (I) was assessed retrospectively in the first questionnaire. Status at two months (II) and status at seven months after infection (III) were prospectively assessed in their corresponding questionnaires distributed at two and seven months, respectively.

### Main outcome variables

The main outcomes were symptoms during the acute phase, presence of symptoms at two- and seven-months follow-up, and chronic fatigue or insomnia at seven-months follow-up.

#### The acute phase of infection

Participants were asked whether they had COVID-19 symptoms (yes/no/uncertain) during the outbreak. Symptomatic or uncertain persons were asked for the presence of specific symptoms (cough, dyspnea, fever > 38 Celsius, muscle pain, joint pain, headache, lethargy, confusion, dizziness, loss of memory, concentration difficulties, loss of taste and smell, loose stools, abdominal pain, chest pain, rash, skin bleeding, tinnitus, loss of hearing and an open-ended question concerning other symptoms). All symptom-related questions had a checkbox as to whether each symptom was still present at the time of answering the questionnaire.

#### Two months follow-up

Participants with symptomatic infection during the acute phase were asked whether they still felt ill at two months when completing the first questionnaire. Answers were dichotomized into symptomatic yes/no, and uncertain participants were categorized as symptomatic at two months if they reported at least one specific symptom and had checked the “still present” checkbox for symptoms during the acute phase, otherwise they were categorized as asymptomatic.

#### Seven months follow-up

All participants, regardless of symptom status during the acute phase and two-months follow-up, were asked whether they experienced any symptoms in relation to the infection (yes, no or uncertain), seven months after. Participants answering yes or uncertain were asked for specific symptoms, omitting the open-ended question. Answers were dichotomized into symptomatic yes/no, with uncertain participants being categorized as currently having symptoms if they reported at least one specific symptom.

#### Fatigue

All participants were asked a single-item question at the two-months follow-up whether they felt fatigued prior to infection, and at both two- and seven-months follow-up whether they currently felt fatigued. If answering yes, participants were asked to quantify the degree of fatigue they experienced during the last 24 h on a sliding visual analogue scale (VAS) from 0 to 100.

The Chalder fatigue questionnaire (FQ) was included at both follow-ups for participants aged 12 years or older. It consists of 13 items of which 11 examine different physical and mental aspects of fatigue [9]. Answers are given on a 4-item Likert scale: Less than usual (0), no more than usual (1), more than usual (2), much more than usual (3). The scores are summarized to give a ‘fatigue score’ (range 0–33), and dichotomized (0, 0, 1, 1) with ‘fatigue’ defined as a dichotomized score of 4 or more. Severe fatigue is present if in addition the ‘fatigue score’ is 23 or more [10]. The last two items on the FQ address the extent and duration of fatigue, and if the fatigue has lasted at least 6 months, it is defined as chronic fatigue (CF) [11]. Missing answers to any of the 11 items were imputed from the mean value of answers among the other respondents for the missing item, if four or less items were missing in total for the respondent in question.

#### Insomnia

The Bergen Insomnia Scale (BIS) [12] was used to assess insomnia at two- and seven-months follow up for all participants, but included in analyses only for respondents aged 12 years or older. BIS is a 6-item questionnaire addressing sleep (questions 1–4) and tiredness (questions 5–6). Answers are given on a 0-to-7-point scale corresponding to number of days in a week during the previous month for which a given item is applicable for the subject. A score of 3 on at least one of questions 1–4 and questions 5–6 was defined as insomnia. Participants who reported subjective sleep problems and met the BIS criteria were defined as insomnia cases.

### Background variables

In the first questionnaire (at two months) participants were asked for their gender, weight and height prior to infection, country of origin and pre-existing comorbidities (diabetes, asthma, allergy, anxiety, depression, rheumatologic disease, COPD, heart disease, hypertension, prior pulmonary embolism or deep vein thrombosis, immunosuppression or chronic renal failure). Age was calculated per April 1st 2021 from the social security number. A child was defined as age below 16 years. Body Mass Index (BMI) was calculated from weight and height, and ISO-BMI [13] was used for children to enable comparison with adults. Participants aged 12 or older were asked about marital status (single, married, cohabitant, divorced or widowed, dichotomized to single or cohabitant), education level (primary school, secondary school, university or college), main occupation (student, worker, self-employed, retired, disabled or unemployed, re-categorized as student, worker or unemployed) and tobacco use (daily or sporadic, dichotomized to yes or no).

### Hospitalization, vaccination, vascular or renal complications

All hospitalizations related to the outbreak were reported and available to the research group, and hence the hospitalization rate was calculated both for the study population and the total population of Ulvik. Participants 12 years or older were asked and electronic patient records reviewed for both COVID-19 vaccination status and renal or vascular complications following infection (cerebral hemorrhage or infarction, transient ischemic attack, deep vein thrombosis, pulmonary embolism or renal failure) both at two- and seven-months follow-up.

### Statistics

Descriptive data were presented as percentages, mean, median and interquartile range (IQR). Pearson’s χ^2^ was used for comparison of proportions between groups with counts of at least 5 or above in each group, and Fisher’s exact for groups with less than 5. The Mann Whitney U-test was used for comparisons of continuous variables. Binary logistic regression was used to calculate odds ratios for binary variable outcomes, and were reported with 95% confidence intervals and p-values. For longitudinal analyses only participants who had answered both questionnaires were included. Statistical analyses were performed using IBM SPSS Statistics for Windows, version 28 (IBM Corp., Armonk, N.Y., USA). Figure 2 was made with SankeyMATIC (www.sankeymatic.com).

## Results

### Study population and comparison with non-responders

The study population comprised 65% (87/134) of infected persons, who consented to participate and responded to the first questionnaire. Characteristics of the study population is shown in Table 1. Four out of 35 (11%) children reported at least one comorbidity, compared to over half of adults (28/52, p = < 0.001). Compared to non-responders, the mean age of responders was higher (29 versus 20 years, *p* = .024) and the proportion of children lower (35/87 vs. 30/47, *p* = .009).

**Table 1:** Baseline characteristics of study population infected with SARS-CoV-2 B.1.1.7 alpha variant in Ulvik, Norway 2021

	All respondents		Symptomatic		Asymptomatic		*p*-value
	*N* = 87		*N* = 60		*N* = 27		
	%	*n*	%	*n*	%	*n*	
**Female**	62	54	67	40	52	14	0.188
**Age**,** y**,** median (IQR)**	31	(9–43)	33	(14–46)	10	(5–34)	0.015*
**Age categories**							
0–15	40	35	30	18	63	17	
16–30	8	7	7	4	11	3	
31–45	30	26	38	23	11	3	
46–60	14	12	17	10	7	2	
> 60	8	7	8	5	7	2	
**Country of origin**							
Norway	77	67	83	50	63	17	
Other European	7	6	5	3	11	3	
Outside Europe	21	14	12	7	26	7	
**Marital status** ^**a**^							
Single	31	18/58	29	14/48	40	4	
Married/cohabitant	69	40/58	71	34/48	60	6	
*Missing*		1					
**Education** ^**a**^							
Primary school	23	12/53	21	9/43	30	3/10	
Secondary school	45	24/53	42	18/43	60	6/10	
University or college	32	17/53	37	16/43	10	1/10	
*Missing*		6					
**Main occupation** ^**a**^							
Student	20	12/59	23	11/48	9	1/11	
Working	63	37/59	81	30/48	64	7/11	
Not working	17	10/59	15	7/48	27	3/11	
**BMI categories** ^**b**^							
Underweight	6	4/64	6	3/49	7	1/15	
Normal	33	21/64	35	17/49	27	4/15	
Pre-obesity	34	22/64	33	16/49	40	6/15	
Obesity, any class	27	17/64	27	13/49	27	4/15	
*Missing*		23					
**Pre-existing comorbidity** ^**c**^							
Any	37	32	45	27	19	5	0.018
Asthma, allergy	20	17	20	12	19	5	
Diabetes	5	4	7	4	0	0	
Hypertension	9	8	13	8	0	0	
Chronic heart disease	8	7	12	7	0	0	
Anxiety and/or depression	9	8	13	8	0	0	
Rheumatic disease	3	3	5	3	0	0	
Chronic renal failure	1	1	12	7	0	0	
Pulmonary embolism	1	1	2	1	0	0	
Immunosuppression	2	2	3	2	0	0	
**Smoker** ^**a**^							
Daily	5	4/58	6	3/47	9	1/11	
Sporadic	5	3/58	4	2/47	9	1/11	
*Missing*		1					
**Severity of illness**							
Hospitalization	8	7/84		-		-	
*Missing*		3					
**Vaccination** ^**d**^							
Cominarty/Pfizer	8	5/59	6	3/48	18	2/11	

^a^ Only answered by persons 12 years or older

^b^ iso-BMI used for persons 17 years or younger

^c^ Zero instances of deep vein thrombosis, COPD, cancer, liver disease and previous stroke

^d^ All five had received one dose by 28.01, and two had received two doses

* OR 1.03 (95% CI: 1.01-1,06), from logistic regression analysis

At the seven-month follow-up, the response rate was 85% (74/87). The mean age of the study population was 32 years (median 33, range 2–98). Participants lost to follow-up were younger (mean age 15 vs. 32 years, *p* = .004), but did not differ significantly from the study population available at seven months by gender or presence or absence of symptoms during the acute phase of infection.

### Symptoms during the acute phase of COVID-19

**Fig. 1:**
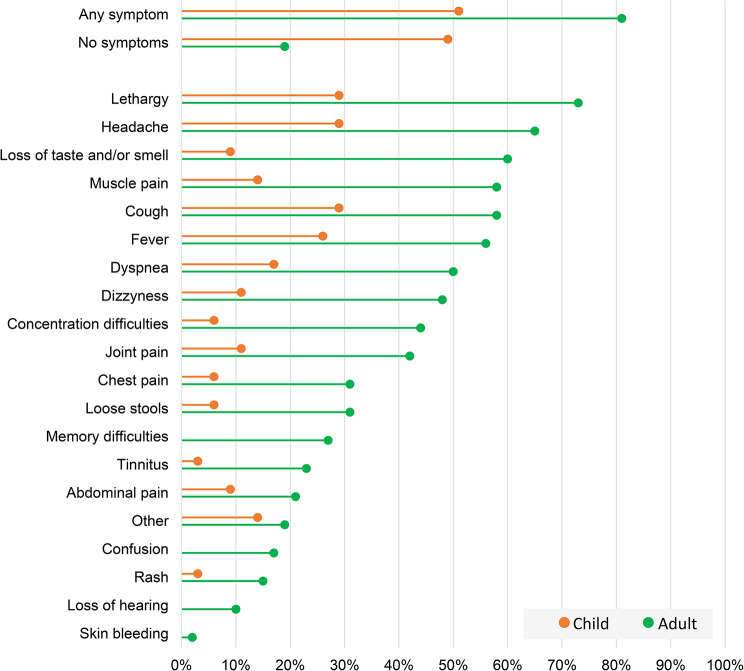
Prevalence of symptoms among *n* = 35 children and *n* = 52 adults in the acute phase of infection during the SARS-CoV-2 B.1.1.7 outbreak in Ulvik, Norway 2021

As shown in Fig. 1, 51% (18/35) of children experienced symptoms during the acute phase, versus 81% (42/52) of adults (*p* = .004).

### Persisting symptoms at two months follow-up

At two-months follow-up 88% (53/60) of participants with symptoms during the acute phase, including all children, reported no longer feeling sick from COVID-19. Among participants still feeling sick, lethargy (6/7) and concentration difficulties (5/7) were the most frequent symptoms. The complete list of reported symptoms at two months post infection is found in Supplementary Table 1.

### Symptom trajectories from the acute phase via two months and through seven months follow-up

**Fig. 2:**
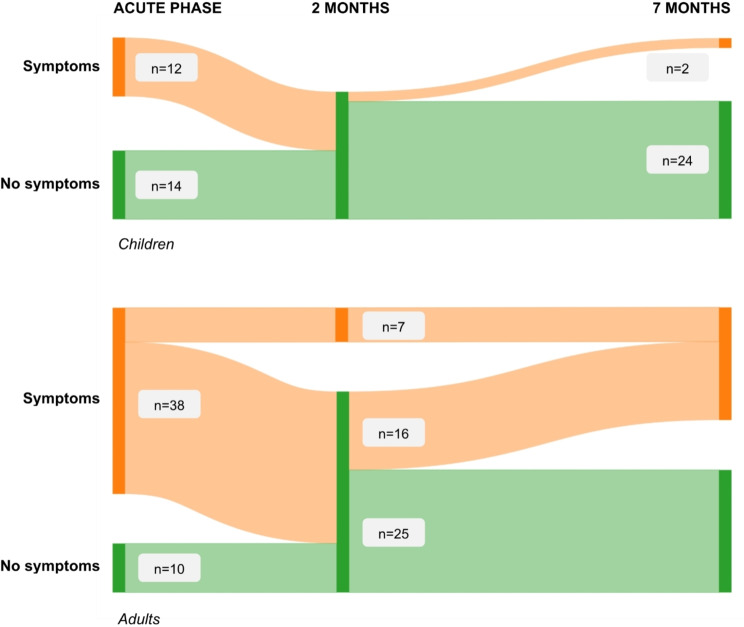
Flow of *n* = 26 children and *n* = 48 adults infected with SARS-CoV-2 B 1.1.7 from initial symptom status, through the study period, Ulvik, Norway 2021

Figure 2 shows the flow of adult and child participants from initial symptomatic status in the acute phase, via two- to seven months follow-up. We had complete data for 74 participants, 26 children and 48 adults. Of all children who were either asymptomatic during the acute phase or had cleared any symptoms by two-months follow-up, 92% (24/26) stayed asymptomatic at seven-months follow-up. For adults the corresponding number was 61% (25/41) of cases.

### Symptoms related to COVID-19 at seven months follow-up

In total, 34% (25/74) reported at least one COVID-19-related symptom seven months after the infection. Among these, 80% (20/25) were female compared to 57% (28/49, *p* = .051) females among participants with no symptoms. The mean age for participants with at least one symptom was 44 years (median 40, range 13–95), compared to 26 (median 16, range 2–98, *p* = .003, OR 1.04 (1.01–1.06)) among those with no symptoms. Only 2/26 children, both teenagers, experienced symptoms versus 48% (23/48, p = < 0.001) of adults. Lethargy and loss of memory were the most frequent symptoms among the 23 adults, reported by 17 and 13 persons respectively. The frequency of all symptoms at seven months is given in Supplementary Table 2. Presence of one or more comorbidities was associated with reporting COVID-19-related symptoms after seven months. Of those with symptoms 52% (13/25) had at least one comorbidity compared to 27% (13/49, *p* = .030) of asymptomatic participants.

### Fatigue

In total, 20% (15/74) of participants, of which only one was a child, felt fatigued at seven-months follow-up, with a VAS-fatigue mean score at 47 (range 18–81). Among participants with symptoms at seven months 52% (13/25) felt fatigued with mean VAS-fatigue score 48 (range 18–81), compared to 4% (2/49, *p* = .001) of participants who reported no symptoms, with mean VAS-fatigue score 42 (range 40–43, *p* = .395 for difference in VAS-fatigue score).

The prevalence of CF at seven months was 15% (8/54), of which four of eight cases had severe CF. Mean VAS-fatigue among CF cases was 55 (range 40–81) compared to 41 (range 18–57, *p* = .082) among non-cases. Among cases of severe CF, mean VAS-fatigue was 59 (range 40–81). Two of the eight CF cases reported feeling fatigued prior to SARS-CoV-2-infection, making the incidence of CF from the outbreak onset through the study period 11% (6/54). Results on fatigue and insomnia variables among participants aged 12 years or older together with their clinical and demographic characteristics are shown in Table 2.

**Table 2:** Characteristics of adults and children 12 years or older (*n* = 54) at 7 months follow up after SARS-CoV-2 B.1.1.7 alpha variant infection in Ulvik, Norway 2021

	Total		Symptomatic at 7 months		Asymptomatic at 7 months		*p*-value	Chronic fatigue-case		Non-case		*p*-value
	*N* = 54		*N* = 25		*N* = 29			*N* = 8		*N* = 45		
	%	*n*	%	*n*	%	*n*		%	*n*	%	*n*	
Female	69	37	80	20	59	17	0.092	88	7	67	30	0.187
Age y (median, range)	41	(37, 12–98)	44	(40, 13–95)	39	(36, 12–98)	.385^a^	54	(48, 30–98)	39	(36, 12–95)	.061^b^
Child < 16y	11	6	8	2	14	4	0.675	0	0	13	6	-
Symptoms during acute phase	80	43	92	23	42	20	0.036	88	7	80	36	0.237
Any comorbidity	44	24	52	13	38	11	0.300	63	5	42	19	0.444
Hospitalization	13	7/53	25	6/24	3	1	0.021	25	2	14	(6/44)	0.291
**Chalder fatigue questionnaire**												
Sum score 2 m mean (median, IQR)	14	(13, 11–17)	17	(16, 13–21)	11	(11, 9–14)	< 0.001	17	(20, 11–26)	13	(13, 11–16)	0.095
Sum score 7 m mean (median, IQR)	15	(14, 11–17)	17	(17, 14–19)	12	(11, 10–15)	< 0.001	23	(23, 17–27)	13	(11–16)	< 0.001
Fatigue case 2 m	44	22/50	71	17/24	19	5/26	< 0.001	71	(5/7)	40	(17/42)	0.219
Fatigue case 7 m	45	24/53	72	18	21	6/28	< 0.001	-		36	16	-
Severe fatigue 2 m	8	4/50	17	4/24	0	0/26	0.046	29	(2/7)	5	(2/42)	0.092
Severe fatigue 7 m	8	4/53	16	4	0	0/28	0.043	50	4	0	0	< 0.001
Chronic fatigue case 7 m	15	8/53	24	6	7	2/28	0.129	-		-		
**Bergen Insomnia Scale**												
Sum score 7 m mean (median, IQR)	11	(11, 3–18)	16	(17, 12–24)	7	(5, 3–12)	< 0.001	15	(14, 8–18)	11	(12, 3–18)	0.410
Insomnia case	40	21/53	67	16/24	17	5	< 0.001	71	(5/7)	36	16	0.072

^a^ OR 1.01 (95% CI: 0.99–1.04), from logistic regression analysis

^b^ OR 1.04 (95% CI: 1.00-1.07), from logistic regression analysis

### Insomnia

The prevalence of insomnia at seven months was 40% (21/53), as shown in Table 2. Insomnia cases were older (mean 48 years) compared to non-cases (mean 36 years, *p* = .039). No significant differences were found between sexes either in mean BIS score (female 12.5 vs. male 8.4, *p* = .124) or proportion of insomnia cases (female 17/36 vs. male 4/17, *p* = .137). The prevalence of insomnia at two months according to BIS was 25% (13/52), compared to 38% (20/52, *p* < .001) at seven months. Among participants with any symptom at seven months, 67% (16/24) represented insomnia cases versus 17% (5/29, *p* < .001) among those without symptoms (Table 2). Most participants with insomnia at seven months (86%, 18/21) had reported symptomatic infection during the acute phase, but this was similar to patients without insomnia (75%, 24/32, *p* = .347). Insomnia at seven months was not associated with having symptoms in the acute phase, but with reporting COVID-19-related symptoms at seven months.

### Hospitalization rate, vaccination, renal or vascular complications

At two months 5/59 were vaccinated against SARS-CoV-2. The same five participants exclusively had received their second dose by seven months follow-up, with an additional 39/54 participants having received their first. No participants under the age of 12 years had been vaccinated against SARS-CoV2 during the study period. The hospitalization rate among all infected persons in Ulvik during the outbreak was 6.7% (9/134), and in the study population it was 8% (7/84), with no deaths attributable to SARS-CoV-2 during the study period. Among the seven hospitalized participants included in the study population, six reported persisting symptoms at follow-up. All were between 31 and 45 years old, 58% (4/7) were female, 43% (3/7) did not have any comorbidities and none had received their first vaccine dose before being infected or hospitalized. Post infectious renal or vascular complications were not reported from any participant neither at two- nor seven-months follow-up.

## Discussion

We present a comprehensive longitudinal study of SARS-CoV-2-infected individuals recruited from an immunologically naïve population in a geographically well-defined outbreak. We found that children underwent asymptomatic infection more often than adults. Most participants, including all children, who were asymptomatic at two-months follow-up stayed asymptomatic during the study period. Among those with persisting COVID-19-related symptoms at seven months, both fatigue and insomnia were common conditions.

### Strengths, limitations

A major strength of the study is the complete, NAAT-verified outbreak from which participants were recruited regardless of age or the presence of COVID-19-related symptoms during the acute phase of infection. With meticulous contact tracing, high testing capacity and rapid sample processing turnaround time it is likely that most cases, if not all, were identified. This enabled a close to true and realistic denominator when assessing the proportion of patients with symptomatic versus asymptomatic infection. To the best of our knowledge, the Ulvik SARS-CoV-2 B.1.1.7 alpha variant outbreak is among the largest, complete clusters of infected individuals from a single source during the COVID-19 pandemic published to date.

Important limitations in this study are the risks of selection and recall biases, and investigation of a small population. Selection bias in COVID-19-research constitutes a major challenge when reporting prevalence data [14], and analyses in a selected subsample of a given population could greatly affect results [15]. The recruitment of participants from a presumably complete group of infected persons in a well-defined geographic and demographic population limits the risk of selection bias, although some still remains due to the responder rate. We did not have information about symptoms present prior to or during infection. If we had been able to control for such data, the load of disease-attributable complaints due to COVID-19 would presumably have been lower [16]. The retrospective assessment of symptoms during the acute phase of infection introduces the risk of recall bias by the study participants or their proxies. Research recruitment using COVID-19 specific messaging also has been shown to appeal to persons with a higher level of concern about the potential severity and effects of SARS-CoV-2 infection [17]. This could at least in part explain why symptoms reported by adults seem to increase from two to seven months follow-up in our study population, although late occurrence of symptoms cannot be excluded by this study.

Non-responders and participants lost to follow-up were younger than the study population, possibly resulting in a greater proportion of symptomatic children in our study. With symptomatic infection and persisting symptoms at follow-up being associated with increasing age and comorbidities, this could skew results towards more sick adults and less sick children. Regardless of the recruitment setting the total number of infected during the outbreak was fairly low and as such some of the analyses were probably underpowered, especially when analyzing the effect of different explanatory variables.

### Comparison to other studies

The constellation of acute phase symptoms in our study population is consistent with the widely accepted perception of COVID-19 [18]. We found that children more often than adults underwent asymptomatic infection with SARS-CoV-2, as has been previously reviewed by Jackson et al. [19] among others. The symptom patterns and frequencies seem similar between adults and children, except for symptoms such as loss of taste and/or smell, dizziness and tinnitus that hold less validity below an age in which subjective reporting would be unfeasible.

#### Fatigue

Fatigue is a common complaint among those with persisting symptoms after COVID-19, as highlighted across a broad swathe of publications, including two recent meta-studies [4, 20] and a review [3]. Our study shows a similar prevalence of reported fatigue among symptomatic respondents. Asking participants about the duration of fatigue and whether it was present prior to infection enabled an estimation of incident CF in Ulvik during the study period. New onset of fatigue after being infected suggests a causal relationship, however our results should be interpreted with caution due to the small number of affected individuals. Whether fatigue is attributable to infection or other pandemic related factors remains complicated to untangle. The prevalence of CF at seven months among participants without symptoms during the outbreak is similar to what has been shown in a normative study in the Norwegian general population [21], possibly reflecting the level of ´background fatigue´ among participants in our study and could suggest that chronic fatigue is related to experiencing persisting symptoms after COVID-19.

A similar proportion of CF cases and non-cases at seven months follow-up appeared to have experienced symptoms during the acute phase of infection. This implies that if CF is related to COVID-19, it is not immediately apparent during the acute phase of infection and the risk of being chronically fatigued could be affected by other factors than having experienced symptomatic infection during the acute phase. A complementing feature is highlighted in a study from southeastern Norway, also during the alpha variant wave, performed among adolescents and young adults who underwent NAAT-testing for SARS-CoV-2. No difference in prevalence of the post COVID-19 condition as defined by the WHO was found between test positive and negatives [22]. A Dutch population-based study with access to pre-infection symptoms among participants in a cohort of SARS-CoV-2 infected also showed that only in 13% of cases matched with non-infected controls could the presence of a core symptom of COVID-19 (of which general tiredness or fatigue was one) be attributed to infection.

Owing to the population profile in Ulvik, there were relatively few young adults between 16 and 30 years both infected (10/134, data not shown) and included in our study population (7/87). In addition, youths attending high school in a neighboring municipality reportedly did not return home for the weekends when the outbreak was at its peak. This age group has been reported to have a high prevalence of fatigue following SARS-CoV-2 infection [23], and the relative absence of this group could bias our results toward lower prevalence of fatigue.

#### Insomnia

The overall mean BIS score is similar to a pre pandemic community normative material from 2008 [12] suggesting no apparent increase in the prevalence of insomnia among infected during the current outbreak. However, among participants who reported COVID-19-related symptoms at seven months a higher BIS score and higher prevalence of insomnia cases is observed compared to those who did not report such symptoms. We are not able to establish whether insomnia in our study population exists as a consequence of the SARS-CoV-2 infection itself, secondary to other COVID-19-related complications, because of pandemic related societal or socioeconomic factors, or if COVID-19 increases the likelihood of insomnia [24]. During the pandemic insomnia has been shown to vary with social invasiveness of lockdown measures [25, 26], and occur more often among persons with comorbidities, anxiety and/or depression and lower age [27]. The lack of similar findings in our population could be due to a relatively healthy population, a small sample size or both.

### Generalizability

We have studied acute COVID-19 and outcomes up to seven months later following infection with one specific variant of SARS-CoV-2 in a defined community setting. The patients are well characterized with both baseline and follow-up data. Consequently, the internal validity is good, and the results should be helpful in the understanding and management of patients in our settings. However, there are limitations regarding the generalizability to other populations or variants of the virus. There are few young adults in our population. Most patients had asymptomatic or relatively mild infection. This might be attributed to the virulence of the particular variant or to the fact that we were able to reach almost the complete population of Ulvik. The population was to a large extent immunologically naïve without prior infection or vaccination, whereas in most settings patients have been exposed several times to different variants of the virus and also to vaccines. The clinical manifestations and immunological mechanisms will often be more complex than is the case in our study, especially when considering post-infectious complications where a range of different factors may play a role.

## Conclusion

In an immunologically naïve population infected with the SARS-CoV-2 VOC B.1.1.7, the clinical features of acute phase symptoms of COVID-19 were similar to previous studies. Children underwent asymptomatic infection more often than adults, and adults more often experienced persisting symptoms. Insomnia and fatigue were common conditions among those with COVID-19-related symptoms seven months after infection.

## Electronic supplementary material

Below is the link to the electronic supplementary material.

Supplementary Material 1

Supplementary Material 2

## Data Availability

The datasets generated and analyzed during the current stydy are not publicly available due to privacy issues arising in the setting of a small community and the number of study participants, but are available as selected data from the corresponding author on reasonable request.
